# Gap junction modulation and its implications for heart function

**DOI:** 10.3389/fphys.2014.00082

**Published:** 2014-02-27

**Authors:** Stefan Kurtenbach, Sarah Kurtenbach, Georg Zoidl

**Affiliations:** ^1^Department of Psychology, Faculty of Health, York UniversityToronto, ON, Canada; ^2^Department of Biology, Faculty of Science, York UniversityToronto, ON, Canada; ^3^Center for Vision Research, York UniversityToronto, ON, Canada

**Keywords:** gap junction communication, connexin, heart, interactome, signaling pathway

## Abstract

Gap junction communication (GJC) mediated by connexins is critical for heart function. To gain insight into the causal relationship of molecular mechanisms of disease pathology, it is important to understand which mechanisms contribute to impairment of gap junctional communication. Here, we present an update on the known modulators of connexins, including various interaction partners, kinases, and signaling cascades. This gap junction network (GJN) can serve as a blueprint for data mining approaches exploring the growing number of publicly available data sets from experimental and clinical studies.

## Gap junction communication in health and disease

Gap junction communication (GJC) describes the electrical and metabolic coupling of cells through specialized cell contacts called gap junctions. In vertebrates, gap junctions are present in most tissues having important roles in development, growth regulation, tissue homeostasis, and communication. They assemble from homo- or hetero-hexameric connexin hemichannels encoded by 20 (rodents) or 21 (human) different genes (Söhl et al., 2005). GJC has been studied in great detail for the last 50 years. These studies emphasized important molecular, biophysical properties, and physiological roles of connexin channels. Other studies revealed connexin structures down to atomic resolution (Maeda et al., 2009; Grosely and Sorgen, 2013) and a multitude of regulatory mechanisms controlling the entire life cycle of these channels from transcription, post-translational modification, to removal of gap junctions and degradation (Laird, 2006; Johnstone et al., 2012a; Su et al., 2012; Thévenin et al., 2013). More recent work demonstrated connexin hemichannel functions under physiological conditions (Bruzzone et al., 2001; Anselmi et al., 2008; Garré et al., 2010) and evidence for channel independent function, e.g., in cell growth and death (Vinken et al., 2012) or migration (Kameritsch et al., 2012). Mutations in connexins were discovered in inherited human diseases like oculodentodigital dysplasia (ODDD, Cx43, GJA1; Huang et al., 2013), X-linked Charcot-Marie-Tooth disease (Cx32, GJB1; Scherer and Kleopa, 2012), Pelizaeus-Merzbacher-like disease or a milder spastic paraplegia (Cx47; Kleopa et al., 2010), Vohwinkel syndrome as well as Keratitis-Icthyosis-Deafness (KID) syndrome (Cx26, GJB2; Lee and White, 2009; Xu and Nicholson, 2013), Erythrokeratodermia variabilis (Cx31, GJB3; Cx30.3, GJB4), Clouston syndrome (Cx30, GJB6) or secondary lymphedema following breast cancer treatment (Cx47, GJC2; Finegold et al., 2012). Furthermore, transcriptional and post-transcriptional alterations and dysfunctional degradation by autophagy (Lichtenstein et al., 2011; Fong et al., 2012) may represent indirect mechanisms causing impaired GJC. Today, a causal relationship, e.g., in the context of seizures (Li et al., 2001; Gajda et al., 2005; Samoilova et al., 2008), cerebral ischemia (Contreras et al., 2004; Talhouk et al., 2008; Orellana et al., 2010), autism (Fatemi et al., 2008), schizophrenia (Meyer et al., 2002; Aleksic et al., 2007), and after trauma (Frantseva et al., 2002) seems plausible. Thus, understanding the exact roles of GJC in health and disease is a highly relevant and timely objective in biomedical and preclinical research. Transcriptome studies have started to provide valuable insight into the consequences of altered connexin expression in animal models (Spray and Iacobas, 2007; Iacobas et al., 2012, 2007a), exploring the use of coordination analysis of gene expression as a strategy to identify connexin related gene networks. The huge amount of transcriptome data available in public databases, together with more sophisticated data processing tools, suggest that investigating transcriptional changes within a physiologically relevant “gap junction network” (GJN) will have wide application potential.

## Modulation of gap junction communication

**Figure 1:**
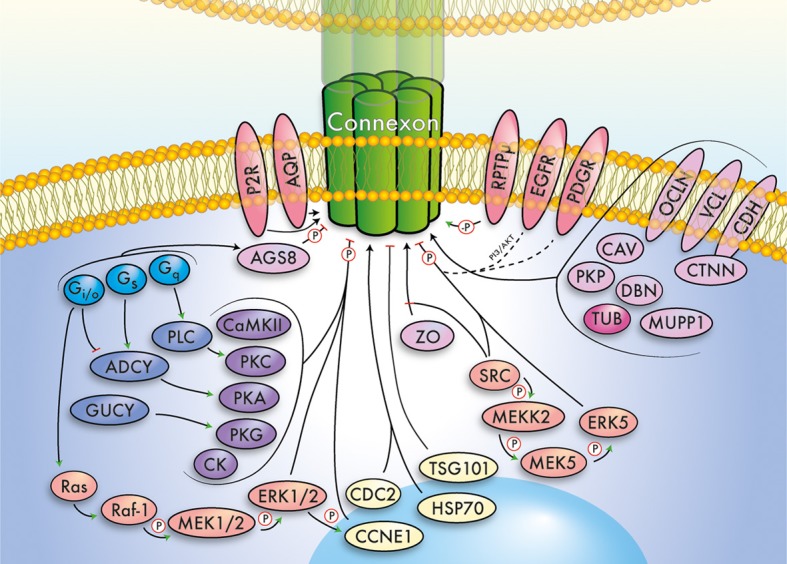
**Simplified summary of the gap junction network.** This cartoon summarizes important signaling pathways, modulators, and interacting proteins of connexins, which converge exemplarily on a (green) connexin gap junction channel. The major functional groups outlined in the main text have been color-coded and relations between groups indicated by arrows. Further, phosphorylation (P) and dephosphorylation (-P) is highlighted. Note that the depicted pathways/interactions will vary for individual connexins. The gap junction network includes G proteins (light blue), cyclases (dark blue), kinases (violet), MAPK/ERK related signaling pathways (orange), receptors (red), scaffolding and junctional proteins (pink), cytoskeleton (dark pink), and cell cycle associated proteins (yellow).

The major cardiac connexin proteins are Cx40 (GJA5), Cx43 (GJA1), and Cx45 (GJC1), having distinct expression patterns and essential roles in propagation of action potentials, metabolic coupling, tissue homeostasis and heart development (Lo, 2000; Nishii et al., 2001; Rohr, 2004; Bernstein and Morley, 2006; Zacchigna et al., 2009; Jansen et al., 2010). Given these important functions, it is not surprising that GJC has been associated with various heart diseases (Jongsma and Wilders, 2000; Severs, 2001; Severs et al., 2004, 2008; Tribulová et al., 2008; Rodríguez-Sinovas et al., 2012; Verheule and Kaese, 2013). Here, we will focus on interacting and modulating proteins, clustered in functional groups, forming the basis for a draft GJN (Figure 1). A complete list of proteins, isoforms, and putative interactions in the GJN can be found in Table 1, a list of proven interactions in Table 2, while functional evidence is presented below. We will not discuss the structurally related, non-gap junction forming pannexins, or LRRC8 (Abascal and Zardoya, 2012), although it is interesting to note that pannexins release cardioprotectants during ischemic events in the heart (Wang et al., 2009; Vessey et al., 2010, 2011; Rodríguez-Sinovas et al., 2012).

**Table 1:** **A draft of a gap junction network gene list**.

ADCY1, ADCY10, ADCY2, ADCY3, ADCY4, ADCY5, ADCY6, ADCY7, ADCY8, ADCY9, AQP1, AQP2, AQP3, AQP4, AQP5, AQP6, AQP7, AQP8, AQP9, AQP10, AQP11, AQP12A, AQP12B, BAX, CALM1, CALM2, CALM3, CAMK1, CAMK1D, CAMK1G, CAMK2A, CAMK2B, CAMK2D, CAMK2G, CAMK4, CASK, CAV1, CAV2, CAV3, CCNE1, CDC2, CDH1, CDH10, CDH11, CDH12, CDH13, CDH14, CDH15, CDH16, CDH17, CDH18, CDH19, CDH2, CDH20, CDH3, CDH4, CDH5, CDH6, CDH7, CDH8, CDH9, CIP85, CSNK1A1, CSNK1A1L, CSNK1D, CSNK1G1, CSNK1G2, CSNK1G3, CSNK2A1, CSNK2A2, CSNK2B, CTNNA1, CTNNA2, CTNNA3, CTNNAL1, CTNNB1, CTNNBL1, CTNND1, CTNND2, DBN1, EGFR, GJA1, GJA10, GJA3, GJA4, GJA5, GJA8, GJA9, GJB1, GJB2, GJB3, GJB4, GJB5, GJB6, GJB7, GJC1, GJC2, GJC3, GJD2, GJD3, GJD4, GJE1, GNA11, GNA12, GNA13, GNA14, GNA15, GNAI1, GNAI2, GNAI3, GNAL, GNAO1, GNAQ, GNAS, GNAT1, GNAT2, GNAT3, GNAZ, GNB1, GNB1L, GNB2, GNB2L1, GNB3, GNB4, GNB5, GNG10, GNG11, GNG12, GNG13, GNG2, GNG3, GNG4, GNG5, GNG7, GNG8, GNGT1, GNGT2, GRB2, GUCY1A2, GUCY1A3, GUCY1B2, GUCY1B3, GUCY2A, GUCY2B, GUCY2C, GUCY2E, HRAS, HSP70-1A, HSP70RY, HSP70-4, HSP70-1B, HSP70T, HSP70-3, KRAS, MAP2K1, MAP2K2, MAP2K3, MAP2K4, MAP2K5, MAP2K6, MAP2K7, MAP3K1, MAP3K10, MAP3K11, MAP3K12, MAP3K13, MAP3K14, MAP3K15, MAP3K2, MAP3K3, MAP3K4, MAP3K5, MAP3K6, MAP3K7, MAP3K8, MAP3K9, MAP4K1, MAP4K2, MAP4K3, MAP4K4, MAP4K5, MAPK1, MAPK10, MAPK11, MAPK12, MAPK13, MAPK14, MAPK15, MAPK3, MAPK4, MAPK6, MAPK7, MAPK8, MAPK9, MLLT4, MPDZ, NRAS, OCLN, P2-receptor, P2RX7, P2RY1, PANX1, PANX2, PANX3, PDGFRA, PDGFRB, PDGFRL, PKP1, PKP2, PKP3, PKP4, PLCB1, PLCB2, PLCB3, PLCB4, PLCD1, PLCD3, PLCD4, PLCE1, PLCG1, PLCG2, PLCH1, PLCH2, PLCL1, PLCL2, PLCZ1, PRKAA1, PRKAA2, PRKAB1, PRKAB2, PRKACA, PRKACB, PRKACG, PRKAG1, PRKAG2, PRKAG3, PRKAR1A, PRKAR1B, PRKAR2A, PRKAR2B, PRKCA, PRKCB, PRKCD, PRKCDBP, PRKCE, PRKCG, PRKCH, PRKCI, PRKCQ, PRKCZ, PRKG1, PRKG2, PTPRM, RAF1, SOS1, SOS2, SRC, TJAP1, TJP1, TJP2, TJP3, TSG101, TUBA1A, TUBA1B, TUBA1C, TUBA3C, TUBA3D, TUBA3E, TUBA4A, TUBA8, TUBAL3, TUBB1, TUBB2A, TUBB2C, TUBB3, TUBB4, TUBB6, TUBD1, TUBE1, TUBG1, TUBG2, VCL

Summarizing GJC/connexin interaction/modulating proteins, we propose a draft of a GJN forming the blueprint to investigate the role of GJC. Known GJC modulators, as well as putative ones (isoforms and other closely related proteins) are included, with the intent to foster research on GJN modulation in health and disease.

**Table 2:** **Summary of connexin interacting proteins**.

**Interacting protein**	**Connexin**	**Type of detection**	**References**
**CELL–CELL JUNCTIONAL AND SCAFFOLDING PROTEINS**			
α-catenin	GJ	co-loc, EM, FRIL	Fujimoto et al., 1997
β-catenin	Cx43	co-loc in cardiac myocytes, β-catenin-IP (N)	Ai et al., 2000; Lee and White, 2009
actin	Cx43	co-loc	Wall et al., 2007; Smyth et al., 2012
AF6	Cx36	AF6-IP (N), Cx36-IP (N), co-loc	Li et al., 2012
AGS8	Cx43	*IVB*, co-loc	Sato et al., 2009
CASK	Cx36	*IVB*, co-loc, Far-WB, CASK-IP (N, RE)	Márquez-Rosado et al., 2012
CAV1	Cx43	CAV1-IP (RE), co-loc, *IVB*	Langlois et al., 2008
CAV2	Cx43	CAV2-IP (RE), co-loc, *IVB*	Langlois et al., 2008
CAV3	Cx43	CAV3-IP (N), co-loc, *IVB*	Liu et al., 2010
CDH1	GJ	co-loc, EM, FRIL	Fujimoto et al., 1997
CDH2	Cx43	N-cadherin-IP (N), co-loc	Lee and White, 2009
drebrin	Cx43	co-loc, *IVB*	Butkevich et al., 2004
MUPP1	Cx36	Cx36-IP (N), MUPP1-IP (N), co-loc	Li et al., 2012
occludin	Cx43	occludin-IP (RE), co-loc	Kojima et al., 1999
p120^ctn^	Cx43	co-loc	Xu et al., 2001
PKP2	Cx43	PKP2-IP (N)	Lee and White, 2009; Sato et al., 2011
tubulin	Cx43	co-loc, *IVB*	Giepmans et al., 2001a,b
vinculin	Cx43	AB-array, Cx43-IP (N)	Iacobas et al., 2007a,b
ZO-1	Cx43	co-loc cardiac myocytes, ZO1-IP (RE, N), Cx43-IP (RE), *IVB*	Giepmans and Moolenaar, 1998; Toyofuku et al., 1998
	Cx45	ZO-1-IP (RE), co-loc, Y2H (PDZ Domain), *IVB*	Kausalya et al., 2001
ZO-2	Cx43	*IVB*, ZO2-IP (N), Cx43-IP (N), co-loc, Far-WB	Singh et al., 2005
ZO-3	Cx45	Y2H (PDZ domains)	Kausalya et al., 2001
**KINASES**			
PKA	Cx35	*IVP*	Ouyang et al., 2005
	Cx36	*IVP*	Urschel et al., 2006
	Cx50	*IVP, in vivo* phosphorylation,	Liu et al., 2011a,b
PKC	Cx43	PKCα-IP (N), Cx43-IP (N; PKCα, PKCε, PKCδ), co-loc (PKCα, PKCε, PKCδ),* IVP* (PKCδ), PKCδ-IP	Bowling et al., 2001; Niger et al., 2010
PKG	Cx35	*IVP*	Patel et al., 2006
PKG	Cx43	*IVP*	Kwak et al., 1995; Patel et al., 2006
CaMKII	Cx43	*IVP*, co-loc	Hund et al., 2008; Huang et al., 2011
calmodulin	Cx32	co-loc	Peracchia et al., 2000
CKI	Cx49	*IVP*	Cheng and Louis, 1999
	Cx43	*IVP*, Cx43-IP (N)	Cooper and Lampe, 2002
CKII	Cx45.6(av)	*IVP, in vivo* phosphorylation	Yin, 2000
MAPK7/ERK5	Cx43	*IVP*, ERK5-IP (RE), Cx43-IP (RE)	Cameron et al., 2003
c-Src	Cx43	Cx43-IP (N, RE)	Toyofuku et al., 2001; Li et al., 2009
CIP85	Cx43	Co-loc, CIP85-IP (RE, N)	Lan et al., 2005
**RECEPTORS**			
RPTPμ	Cx43	RPTPμ-IP (RE), Cx43-IP (N, RE)	Giepmans et al., 2003
AQP0	Cx45.6(av)	Cx45.6-IP (N), co-loc	Yu and Jiang, 2004; Yu et al., 2005
	Cx56(av)	Cx56-IP (N)	Yu and Jiang, 2004
P2X_7_	Cx43	Cx43-IP (N), P2X_7_ (N), co-loc, AB-array,	Fortes et al., 2004; Iacobas et al., 2007a,b
**CELL CYCLE/CELL DEATH**			
cyclin E	Cx43	co-loc, Cx43-IP (N), cyclin E-IP (N)	Johnstone et al., 2012a,b
HSP70	Cx43	co-loc, Cx43-IP (N)	Hatakeyama et al., 2013
TGS101	Cx43	Y2H, TGS101-IP (N), co-loc	Auth et al., 2009
	Cx36	Y2H, TGS101-IP (N)	Auth et al., 2009
	Cx30.2	Y2H	Auth et al., 2009
BAX	Cx43	Cx43-IP (RE)	Sun et al., 2012

Summary of connexin interacting proteins. This table summarizes documented interactions described in the text and the detection methods used. It does not include indirect interactions with regulatory pathways. Abbreviations in alphabetic order: AB-array, antibody array; av, avian connexin; co-loc, co-localization in cells or tissues; IVB, in vitro binding, binding of peptides or functional domains; Far-WB, Far western blot; IVP, in vitro phosphorylation; N, native, non-transfected tissues, cells, or cell lines; RE, one or both IP partners were expressed in recombinant cells; Y2H, yeast two hybrid assay.

### Cell-cell junctional and scaffolding proteins

A shared communality among connexins is the binding to junctional, scaffolding and cytoskeletal/transport proteins. Interactions between connexins and the tight junction proteins ZO-1, ZO-2, and ZO-3 (TJP1, TJP2, TJP3) vary regarding different connexin and ZO proteins (Giepmans and Moolenaar, 1998; Toyofuku et al., 1998; Kausalya et al., 2001), regulating connexon to gap junction transition (Rhett et al., 2011) and, as shown for ZO-1, can be regulated by c-Src in cardiac myocytes (Toyofuku et al., 2001). Increased interaction of ZO-1 with Cx43 plays a role in Cx43 down-regulation and reduced Cx43 gap junction size in congestive heart failure (Bruce et al., 2008). Cell adhesion proteins like E-cadherin (CDH1) and α-catenin are co-localized in newly formed gap junctions (Fujimoto et al., 1997), and E-cadherin mediated cell–cell contacts were shown to increase GJC (Jongen et al., 1991). p120^ctn^ (CTNND1) (Xu et al., 2001) and β-catenin (CTNNB1) (Ai et al., 2000) also co-localize with Cx43, and Cx43 was further found to immunoprecipitate with β-catenin (Li et al., 2009). N-cadherin (CDH2)/connexin interactions were also reported (Li et al., 2009). CDH2 antibodies inhibit gap junction formation (Meyer et al., 1992), and cardiac specific CDH2 knockout in mice causes reduced GJC and sudden death (Li et al., 2005). Vinculin (VCL) interacts with connexins (Iacobas et al., 2007b), and cardiac myocyte specific VCL knockout caused Cx43 dislocation, dilated cardiomyopathy, and sudden death (Zemljic-Harpf et al., 2007). VCL also binds directly to ZO-1, stabilizing gap junctions in the heart (Zemljic-Harpf et al., 2014). The tight junction protein occludin (OCLN) was shown to interact with Cx32 (Kojima et al., 1999) and ZO-1 as well as ZO-2 (Furuse, 1994; Itoh et al., 1999).

AGS8 (FNDC1) forms a scaffold for G_βγ_ subunits and Cx43 and elicits phosphorylation and subsequent internalization, an effect involved in hypoxia-induced apoptosis in cardiomyocytes (Sato et al., 2009). In the brain, the scaffolding proteins MUPP1 (MPDZ) and AF6 (MLLT4) interact with Cx36 (Li et al., 2012). Membrane targeting, cellular migration and wound healing are modulated by Cx43 and interaction with the multidomain scaffolding protein CASK (Márquez-Rosado et al., 2012). Further, all three known human caveolins (CAV), a group of proteins found in lipid rafts and the membrane, interact with Cx43 (Langlois et al., 2008; Liu et al., 2010), increasing GJC (shown for CAV1 and CAV2). Drebrin (DBN1) interacts with Cx43 maintaining Cx43-containing gap junctions in their functional state (Butkevich et al., 2004), likely involving further interactions with the cytoskeleton.

### Cytoskeleton

Connexins are known to directly interact with α-and β-tubulin (Giepmans et al., 2001a,b). There are multiple different tubulin subunits and regional differences in their expression may be linked to schizophrenia (Moehle et al., 2012). Direct interactions of connexins with actin were not reported, but connexins co-localize with actin, which was linked to anterograde Cx43 trafficking (Wall et al., 2007; Smyth et al., 2012). Interactions with actin can be mediated via various scaffolding proteins including drebrin (Butkevich et al., 2004; Majoul et al., 2007) and ZO-1 (Rhett et al., 2011). Further, Cx43 interacts with plakophilin-2 (PKP2) (Li et al., 2009; Sato et al., 2011), a protein linking cadherins to intermediate filaments in the cytoskeleton. Finally, expression of six cytoskeletal proteins (actin, tropomyosin, microtubule-associated protein RP/EB1, transgelin, GFAP, cofilin-1) were differentially regulated when Cx43 expression was targeted in astrocytes with small interfering (si)RNAs (Olk et al., 2010).

### Kinases

Phosphorylation of connexins has various effects on GJC and plays major roles at several steps of the connexin lifecycle, including trafficking, assembly/disassembly, degradation, and gating (Lampe and Lau, 2004). PKA can phosphorylate connexins and promote their synthesis and assembly/stability (Imanaga et al., 2004; Ouyang et al., 2005; Zhang et al., 2005; Urschel et al., 2006; Liu et al., 2011a). PKCs (PRKC) modulate Cx43, including direct phosphorylation through PKCε (PRKCE), and increased phosphorylation mediated by PKCα (PRKCA) (Bowling et al., 2001). Further, PKCδ (PRKCD) was shown to bind to Cx43 (Niger et al., 2010). PKCs are considered a therapeutic target due to the expression of multiple PKCs in the heart and their expression changes and contribution to heart diseases (Liu et al., 2009; Palaniyandi et al., 2009). The cGMP dependent Protein kinase G (cGK, PKG, PRKG) was also reported to phosphorylate connexins and modulate their expression (Kwak et al., 1995; Patel et al., 2006; Joshi et al., 2012). Mammals inherit two PRKGs, cGKI (PRKG1), and cGKII (PRKG2), where PRKG1 is the main PRK in the heart. PRKG1 has well-known functions in the cardiovascular system, including excitation-contraction coupling, contractility, CM hypertrophic remodeling and more, where elevated cGMP levels protect against adverse ventricular remodeling (Balligand and Hammond, 2013; Frantz et al., 2013). In the failing human heart, PKA, as well as PKC and PKG, can phosphorylate cardiac ryanodine receptors, resulting in defective channel function due to increased sensitivity (Takasago et al., 1991; Marx et al., 2000). Ca^2+^/calmodulin-dependent protein kinase II (CaMKII) can phosphorylate Cx43, and its activation and/or increased expression occurs in cardiac disease states like infarction, hypertrophy, and myocardial ischemia (see Erickson and Anderson, 2008; Huang et al., 2011 and references within) and is therefore considered a drug target in heart failure (Bers, 2010). The δ (CAMK2D) subunit is the highest expressed CaMKII in the heart, besides the γ (CAMK2G) subunit being expressed at lower levels (Schworer et al., 1993; Edman and Schulman, 1994). Calmodulin (CaM) activates CaMKII, and also directly modulates connexin gating properties and mediating Ca^2+^-induced uncoupling of gap junctions (review: Zou et al., 2014). Connexins can be modulated by casein kinase 1 (CK1) and CK2 (Cheng and Louis, 1999; Yin, 2000). Besides the finding that CK1δ (CSNK1D) regulates Cx43 gap junction assembly (Cooper and Lampe, 2002), little is known about which CKs targets for other connexins. CK2α1 dependent phosphorylation may be involved in the development of cardiac hypertrophy (Eom et al., 2011).

### Map kinase signaling cascades

The mitogen-activated protein kinase (MAPK) cascades are key intracellular signaling pathways regulating diverse cellular functions such as proliferation, differentiation, survival, development, stress response, and apoptosis. Multiple MAPK cascades have been identified, and although often described as linear, they display significant cross talk (Keshet and Seger, 2010). In the heart, H-Ras, K-Ras, and N-Ras are expressed (Potenza et al., 2005). MAPKs have functions in heart development and are also involved in heart disease formation (Rose et al., 2010). MAPK phosphorylation of connexins is well-documented (reviews: Giepmans, 2004; Solan and Lampe, 2005), e.g., MAPK7/ERK5 was reported to phosphorylate and associate with Cx43, regulating gap junction uncoupling (Cameron et al., 2003). The non-receptor protein tyrosine kinase protein c-Src inhibits the interaction of Cx43 and ZO-1 in cardiac myocytes (Toyofuku et al., 2001). Further, c-Src activation was shown to inhibit gap junctional coupling and remodeling in ischemic heart disease (review: Giepmans, 2004; Rutledge et al., 2012). A Rab-GAP-like protein, CIP85, interacts with Cx43 and induce its internalization and degradation (Lan et al., 2005; Cochrane et al., 2013).

### Heterotrimeric G-proteins

G proteins can interact with GJC by their activation/inhibition of different signaling cascades, e.g., via adenylyl cyclase or phospholipase C (see below). General consent is that GNAI2 is the main G_iα_ in the heart, GNAI3 is expressed in lower amounts and GNAI1 is not expressed (Eschenhagen et al., 1992). However, there are few studies investigating expression of GNAI1 in detail. One newer study reports some cardiac GNAI1 expression (Dizayee et al., 2011) in the heart, alongside the knowledge of its expression in erythrocytes (Olearczyk et al., 2004) and thrombocytes (Patel et al., 2003). GNAI2 is thought to be up-regulated in various heart diseases, but maybe not in ischemic heart disease (ICM) (Feldman et al., 1988; Neumann et al., 1988; Böhm et al., 1990; Eschenhagen et al., 1992). Lack of G_αo_ leads to tachycardia and defects in short-term heart rate dynamics (Zuberi et al., 2008). G_o_ and G_i_ may be involved in gap junction assembly, as pertussis toxin (PTX) sensitive G proteins were linked to Cx43 trafficking (Lampe et al., 2001). Overexpression of G_s_(GNAS) causes many features of dilated cardiomyopathy (DCM) (Iwase et al., 1997), and haplotypes causing different expression levels of G_s_have been found in humans (Frey et al., 2009), providing a putative link to heart disease risk. G_q_ (GNAQ) overexpression leads to heart hypertrophy and contractile failure in transgenic mice (D'Angelo et al., 1997; Fan et al., 2005), and knockout prevents ventricular hypertrophy in response to pressure-overload (Wettschureck et al., 2001). G_α13_ regulates the expression of hypertrophic and fibrotic genes in cardiomyocytes, and inactivation prevents cardiac decompensation (Finn, 1999; Takefuji et al., 2012).

### Cyclases and phospholipase C

Modulators of the soluble guanylate cyclase (sGC, GUCY) are promising new drugs for heart failure treatment (Mitrovic et al., 2011). sGC is a heterodimer composed of one α (GUCYA), and one heme-binding β domain (GUCYB), of which sGCα_1_β_1_ is the principal heteromer in the heart (see Mitrovic et al., 2011 and references within). Adenylyl cyclase type III (ADCY) is considered a therapeutic target for heart diseases, where from 10 known ADCYs ADCY5 and ADCY6 are the predominant ones in the heart, expressed in a development-dependent way (e.g., Feldman, 2002 and references within), but several others are also expressed (Ludwig and Seuwen, 2002). Phospholipase C (PLC) cleaves phosphatidylinositol 4,5-bisphosphate (PIP_2_) into DAG and inositol 1,4,5-trisphosphate (IP_3_). DAG remains bound to the membrane, and IP_3_ is released as a soluble structure into the cytosol activating IP_3_ calcium channels in the smooth endoplasmic reticulum. In addition, calcium and DAG activate PKC. A majority of the 15 known PLCs is present in the heart and some were linked to heart dysfunction (Schwertz and Halverson, 1992; Meij et al., 1997; Hwang et al., 2004; Mangat et al., 2006; Ichise et al., 2009; Otaegui et al., 2010). PLCβ3 was reported to co-localize with Cx43, via the scaffolding protein ZO-1 (see below), where localized changes in PIP_2_ levels dictate channel inhibition (Van Zeijl et al., 2007).

### Receptors

Connexins interact with various other membrane proteins. The receptor protein tyrosine phosphatase (PTP) family regulates a variety of cellular processes including cell growth, differentiation, and mitotic cycle. RPTPμ (PTPRM) can bind and possibly dephosphorylate Cx43, counteracting c-Src phosphorylation, and preventing channel closure (Giepmans et al., 2003). The epidermal growth factor receptor (EGFR) is a receptor tyrosine kinase of the ErbB family. EGFR activation led to connexin phosphorylation and increased cytosolic localization of Cx43 possibly via the PI3/Akt signaling pathway (Díez et al., 1998; Abdelmohsen et al., 2003; Dubé et al., 2012). Platelet-derived growth factor receptors (PDGFRs) are receptors with intracellular tyrosine kinase activity, initiating intracellular signaling through the MAPK, PI3K, and PKCγ pathways. PDGFR activation was shown to lead to Cx43 phosphorylation by MAPK signaling (Hossain et al., 1999a,b, 1998a,b; Shen et al., 2013). PDGFRs have a vital role to load-induced cardiac stress response, angiogenesis, and regeneration (Schatteman et al., 1995; Van den Akker et al., 2008; Bleyl et al., 2010; Chintalgattu et al., 2010; Kim et al., 2010; Chong et al., 2013). The cystic fibrosis transmembrane conductance regulator (CFTR) regulates GJC possibly via a complex mechanism involving c-Src, modulating voltage sensitivity and gating. Further, functional interaction of gap junctions, CFTR and glutamate receptors (GluRs) were reported, although the molecular mechanism is unclear (review: Chanson et al., 2007). GluRs were found in the human myocardium, conducting system, nerve fibers, and intramural ganglia cells (Gill et al., 2007), and glutamate changes intracellular calcium oscillations in cultured rat myocardial cells (Winter and Baker, 1995). Together, GluRs are likely to play a physiological role in heart functions including contraction and rhythm, although their precise role is still elusive. Various aquaporins (AQP) are expressed in the heart, and although the information available is still limited, they were reported to mediate water flux across endothelial membranes, modulate calcium signaling, and nutrient delivery to the heart (Rutkovskiy et al., 2013). AQP0 was shown to interact with gap junctions and in particular with Cx50 in differentiating lens fibers (Yu and Jiang, 2004), enhancing gap junctional coupling (Liu et al., 2011b), suggesting a putative role for AQP/connexin interactions in the heart. Finally, interactions between connexins and purinergic receptors provide an interesting link of connexins to ATP signaling (Fortes et al., 2004; Iacobas et al., 2007b).

### Cell cycle/cell death

Beyond interactions at the plasma membrane and cytosol, connexins can interact with proteins shuttling between cytoplasm and nucleus, or proteins located in mitochondria. Cx43 interacts with cyclin E (CCNE1), for example after MAPK phosphorylation, promoting smooth muscle cell proliferation (Johnstone et al., 2012b). Cx43 also competes with cyclin D1 for binding to heat shock protein 70 (HSP70) (Hatakeyama et al., 2013). Further, degradation of connexins was linked to binding to tumor susceptibility gene 101 (TSG101), an ubiquitin–conjugating enzyme associated with the cell cycle, turnover of proteins, and transcriptional regulation (Auth et al., 2009). Cyclin-dependent kinase 2 (CDC2) was shown to phosphorylate Cx43 in a cell-cycle dependent manner (Kanemitsu et al., 1998; Lampe et al., 1998). Connexins also interact with BAX, a member of the Bcl-2 protein family located in the outer mitochondrial membrane, to regulate apoptosis (Sun et al., 2012).

## Future directions: toward meta-analysis of the gap junction network?

Experimental investigation of the GJN is challenging, due to the large number of putative interactions, procedural issues or the huge experimental variations caused by small sample sizes frequently found in studies using human tissues. However, meta-analyses can capitalize from the growing number of multiple microarray and other “–omics” studies publicly available. Technically, different approaches to merge and perform a statistical analysis have been established and various software tools allow users to process microarray data (Saeed et al., 2003; Gentleman et al., 2004; Reich et al., 2006; Tseng et al., 2012; Xia et al., 2013). Unfortunately, cross-comparison of studies is still a major challenge, but the recently developed online platform INMEX (Xia et al., 2013), or a LabVIEW-based software tool called Array Data Extractor (ADE) (Kurtenbach et al., 2013) are efforts toward making microarray data available in a user-friendly way to a large community. This opens the opportunity to test physiologically relevant changes of the proposed GJN in health and disease.

### Conflict of interest statement

The authors declare that the research was conducted in the absence of any commercial or financial relationships that could be construed as a potential conflict of interest.
